# Reports on Obstetrics and Diseases of Women

**Published:** 1873-04-15

**Authors:** S. H. Birney

**Affiliations:** Urbana, Ill.


### REPORT ON OBSTETRICS AND DISEASES OF WOMEN.

AN ESSAY READ BEFORE THE CENTRAL ILLINOIS MEDICAL SOCIETY, BY S. H. BIRNEY, M. D., OF URBANA, ILL.

Your Committee, in making their report upon Diseases of Women, and Obstetrics, have thought best not to be sternly restricted to any methodical order, but to submit a report of such cases as will be most likely to furnish the greatest amount of interest and practical information, assuming that all diseases accumulate or increase in interest proportionally as they are of frequent occurrence.

Of all the various callings or professions of life, none so imperatively demand a clear head, a self-reliant, thinking, practical mind, as the profession of medicine; and more particularly so at this present age, in the special branches. From the days of Celsus, in no period has the practice been so difficult, unsettled, and complicated, as at the present; not only in the practice of medicine, in the strictly modern meaning of that term, but diseases of women, and obstetrics as well. In the early history of medicine, the simple theories and remedial appliances had but little influence either in aiding or opposing the efforts of nature. The present age is rife with theories; full to repletion with means and appliances; theories speculative, antagonistic and dangerous; in some instances aiding, in others thwarting every effort and purpose of nature; remedies potent, harmless, and poisonous; and of these latter, their name is legion.

Is it possible for any one to enter this vast field of chaos, in search of truth, without a mind and will to think and act for himself?

But perhaps a glance at the theories and practices of the present time will better elucidate the many difficulties the physician has to encounter. The great ambition of the physician of the modern age is alone gratified when he gives free scope to his mental power in the development of some new theory, disease, instrument, or remedy; especially in diseases of women, and obstetrics. And is it not the observation of every physician, that, whether actuated with a desire to emulate either himself or the profession, this undue effort tends more largely to the obstructing of the medical pathway than to make plain and practical the duties of the physician? As an instance of this fact, you will pardon me for referring to the observation, made by a distinguished European author to his class. He says: "The usual curriculum of study given in the schools is sufficient to enable any one with ordinary practical ability to diagnose all common or ordinary diseases; but the detecting of diseases peculiar to women, in their complicated and obscure shades, furnishes a condition involving questions of the most intricate and important nature." These teachings are well intended to inculcate impressions perfectly erroneous and false.

Now, it is true that the distinctive peculiarities of the female constitution have, from the earliest memory of the healing-art, in every important era in the history of the great past, and perhaps for all time to come, will be regarded of peculiar interest, and may be well worthy especial consideration and attention. But after all these long years of toil, of the most earnest study and microscopic inquiry, laboring constantly and incessantly to bring before the medical world some peculiar, unknown pathological condition common alone to the uterine organs, we are left to retrace the stream of medical science, over which we have been so unconsciously gliding, back to the anatomist and physiologist, to inquire of them what *is* there peculiar or different in the structure or phenomena of these organs to necessitate such unlimited straining and labor? What *are* these peculiar diseases referred to, masked in shades obscure? Is it *true* that the arteries, veins, nerves, lymphatics, or any part of the whole parenchymatious tissue of the uterine system differ so materially in their anatomical structure in the uterus from that of any other part of the body as to make it subject to diseases peculiar to itself? That diseases of the uterus seem at times to be disguised in shades obscure and difficult of detection, no one will pretend to deny; and neither will it be denied that all other organic lesions of the female body are at times equally involved in obscurity. But to attempt to account for these anomalous conditions upon the hypothesis of there being a disease peculiar to that organ not well understood, as the learned gentlemen would have us believe, either in pathology or treatment, only to physicians of metropolitan fame, is assuming a position, to say the least of it, not entitled to much credulity from physicians of reading and bedside observation. The deep anxiety, the earnest solicitude and care which has actuated the earnest physician to spend many a long and tedious hour at the bedside, interpreting these morbid and distrustful phenomena; the terrible writhings and contortions of every muscle of the body, or, indeed, any of the protean forms of hysteria, inclusive of the false phases alluded to, asks no better solution of their true nature than that there is predominating within woman a delicate, *finely-developed*, *highly excitable nervous* condition, and in consequence of which, alone, she is largely predisposed to *diseases* of a *nervous* character, and not so much to mysterious and not well-understood organic diseases; and the uterus being a very vascular and highly nervous organ, when diseased, through its direct relationship with the great sympathetic nervous system, would very naturally call into sympathy other members of the same body; indeed, no fact is more evident to the practitioner than that it is capable of exercising a dread and baleful influence on every organ in the body, no matter how remote from the true seat of the disease. And not only does it extend its influence throughout her physical organization, but in many instances her mind, body, and soul suffer simultaneously; and herein consists the great and only difficulty, if indeed it must be so regarded, in the diagnosis and treatment of diseases belonging to this class. But, ever holding before the mind the various pathological conditions and phenomena likely to result from, and as, perhaps, we might say, are natural and inevitable sequences of diseases of the uterus, together with a careful and vigorous study of the temperament, idiosyncracy habits of life, nature, course and termination of previous diseases, if any, these anomalous conditions will be abridged, and in many instances make a plain and practical pathway. Diseases of the uterus are, in our opinion, as well if not much better understood, as a rule, than lesions in any other organ in the body. In all organic diseases we are apt to expect and look for a common, definite, and invariable train of phenomena. These expectations always have been, and ever will be, met with disappointment. The ever-varying phases of diseases of the uterus are, of all other organic lesions, the best calculated to attract the mind to organs entirely remote from the true seat of disease. Indeed, in no two instances have we ever found diseases of this organ indicated by the same train of symptoms. The nervous system succeeds perhaps more completely in perfecting the action of reflex sympathetic sensation than in any other organ; in many instances, all its diseased phenomena, even pain and disease itself, are reflected to, and established in, remote organs; and, indeed, in many instances which have been brought before our notice, these secondary diseases resulting from the remarkable sympathy, involved more structure, and ultimately assumed a vastly more serious character than in the primary lesions. A case in practice will, perhaps, better elucidate this fact. From my note-book of practice, in the year 1864, the following case is substantially taken. In the month of April of that year I was called to see a lady supposed to be dying of phthisis pulmonalis. Arriving at the house, and upon entering the sick chamber, I saw at a glance that the physical signs indicated the existence of this morbid condition in its most serious form. Interrogation elicited from the patient the following statement: "Am forty-five years old; have five living children; miscarried last conception—time, three months; have never had any indications of disease until about one year ago. The first symptom at this time was extreme fatigue after moderate exercise. This was succeeded in a short time with shortness of breath, and dry, hacking cough. This condition, in three months' time, developed into a cough, very harrassing. Expectoration free and copious; have been taking medicine for the past six months from five different physicians, some of merit, ability, and practice. From the greater and more learned part was treated for chronic bronchitis. Have taken no medicine for the past three months, except expectorant, being at times alternately better and worse."

A careful examination revealed the following present condition: Cough incessant; expectoration very profuse, of a thick, whitish consistency; respiration hurried. By auscultation, the mucous rale could be distinctly heard over both lungs; pulse 110; no fever; anorexia; great emaciation; pale and anemic; dejected as to all future hopes. Has had slight mucous discharges from her vaginum for the last two years. No hereditary tendency; temperament well marked, nervous sanguine.

The peculiar manner of the inception of her disease; extreme exhaustion after moderate exercise; the abortion, and vaginal discharges, at once suggested the probable existence of diseased womb. These convictions were stated to the patient, and permission asked to use the speculum, which was readily granted, the use of which disclosed the existence of a very unsuspected malady on the part of the patient, and made very plain the true seat of the disease. The os uteri was dilated sufficient to admit the end of index and middle finger, hypertrophied, and of a pale, bloodless appearance. A very large ulcer occupied well nigh the entire right half of the os, and extended well up the canal, of a fungus character. It certainly afforded great pleasure to inform this lady that her case was not beyond the reach of remedies, and that the strongest hope was entertained of a complete restoration of perfect health, as there could be nothing more evident than that the lung trouble, anemia, and general emaciation, were but the sequel to this primarily diseased uterus. In proof of the correctness of this conclusion, the treatment and result is herein submitted. Internal treatment, one pill of quinine and Vallett's ferruginous mass, in proportions of one grain of the former and two of the latter, was ordered every six hours. Also,

℞.—Tinct. ferri chlor., ʒ ii.

Syrup zinziber, ℥ i.

Syrup simplex, ℥ ii.

Dose.—Teaspoonful every six hours; nourishment *ad interim.*

To the ulcer a topical application was first made of penciled nitrate of silver, applying it well up the cervix, to be followed and continued twice a day, with free injections of lukewarm water medicated with saccharum saturine, and opii, pulverized, until next visit. Early in morning of following day was sent for in great haste; patient supposed to be dying. Arriving at the house, patient was found in a truly critical condition; all former described symptoms greatly intensified; breathing labored; pulse scarcely perceptible; cold, clammy sweat all over the body. morphine was ordered sufficient to quiet nervous irritability; beef essence and brandy were to be given, in addition to the above, every two hours. From this to the fourth day patient rallied, at which time acid nitrate of mercury, was topically applied to the ulcer, and followed, as before, with free ablutions. This application was repeated on the sixth, twelfth, and eighteenth days, after which penciled silver nitras was used for a period of six weeks, according to indications, followed with injections, and internal treatment as above. At the end of the seventh week, patient was so far recovered as to justify the withdrawal of the use of all medicines, and no other treatment was from this time forward used, excepting the internal use of iron, and the free ablution per vaginum, the water used being strongly medicated with ferric alum, which we regard as an indispensable remedy in the treatment of these affections.

On the fourth month, dating time from first visit, to the great surprise of all interested, patient was discharged, free from cough, and in the enjoyment of her former perfect health, and has continued so to this present time.

What are the important facts indicated in the history of this case?

First, it proves most conclusively that there is a remarkable sympathy existing between the uterus and other organs, in disease, and the still more remarkable and deceptive properties of its nervous tissue in transmitting its diseased phenomena, and the efficiency with which its actions are endowed in establishing even disease itself, of the most grave and serious character, in organs entirely remote.

Another important fact elucidated in this case is the importance of examining diseased conditions with a view of ascertaining facts, rather than to carry out some finely spun theory; and that when diseases of the womb are examined as they should, and may be, although seemingly of the most intricate character, are, in the majority of instances, made plain, simple, and practical, and, of all other diseases, the most kind in yielding to remedial appliances.

Another important fact is the non-reliability of certain symptoms which, from time immemorial, have been held as essential to the existence of this malady, not one of which existed in this case, not even so much as a well-established leucorrhœa. The last, yet not by any means the least other fact, which we would call attention to in this case, is the utility of the speculum in the examination of this class of diseases, about which so much has been said and written, by doctors of both continental and European fame, some supporting it, others holding its use to be unchaste, indelicate, and wholly unnecessary, inasmuch as they claim that examinations made by the touch are equally reliable and much more agreeable to the delicate and sensitive female. A preference for the position of the patient, held as essential in this form of examination, though oftimes rude and irritating; manipulations actually necessary to make an expert in the use of the touch in detecting the disease and the application of remedies thereto, is a modesty pitiable and unenviable in the extreme, and, in our opinion, exists much more largely in the false imaginations of the physician, than with the patient.

If the latter assumption is true, that it is equally reliable, then it is true that the treatment of ulcers, and, in fact, any disease of the external surface of the body, would be better treated, and their pathological conditions better, or as well understood, by excluding the sense of sight altogether. But, admitting that instances do occur in which the diagnosis and treatment of diseases of this organ may be effectual by these means, are there not instances in which it is totally inadequate and unreliable?

Take, for instance, the two following cases: In the spring of 1861 I was called to a lady aged 64, of Irish descent, and of the common national, nervous temperament; had given birth to nine healthy, living children; had had two premature deliveries; had never suffered from sickness until within the last month. The first indication of *her* disease, using her own language, was a fluttering sensation, a little inferior and to the right of the umbilicus, being the right iliac. In short, there were no other indications of disease, at this time, excepting occasional nausea and vomiting. She sternly disclaimed ever having had any previous disease of either bowels, kidneys, urinary organs, or uterus. Menstruation had ceased some ten years; had always been healthy, and in no instance followed with unhealthy discharges. Finding no well-marked indication of disease of any of the other viscera of the bowels, a diseased uterus was supposed to be the most likely cause of *this* anomalous condition, and consequently an examination was instituted, per vaginum, by the touch, no better means being at hand, hoping to be at once relieved of all uncertainty in the diagnosis of *her* disease. This expectation soon met with sad disappointment, as the touch furnished no evidence of disease whatever, excepting a very slight induration of the os, which condition we did, and *do* not, regard as necessarily incompatible to, nor uncommon in perfect health. Relying implicitly at this time upon this method of examination, the impression first made of the probable existence of uterine disease was at once dismissed, and for the present decided to treat present indications. A laxative enema was prescribed, to be followed with an anodyne enema. This, however, and in fact all other remedies used furnished but temporary relief. Three weeks later, patient no better. Nausea and vomiting, which at first demanded little attention, have now assumed grave and unmanageable proportions, rapidly ejecting both nourishment and remedies. This, together with the general prostration and emaciation, presented the most important and alarming phase of the disease. The only means from which relief could now be obtained was blistering and the hypodermic use of morphine; and even these were far short of accomplishing what was desired. My esteemed and worthy friend, Dr. Winston Somers, was called in consultation. A very thorough and careful examination was made according to Decosta's method of exclusion. Finally, a very indefinite and unsatisfactory conclusion was arrived at, in favor of the probable existence of cancer of pyloric orifice of the stomach. On the following morning a suspicion once more arose respecting the uterus, and of the possible inefficiency of the former method of examination, and to set this question forever at rest, the speculum was brought into use. Before using it, however, an examination was again made by taxis, and resulted as before, without having the least apprehension aroused of disease in that region. A bivalve speculum was then introduced, and to my great surprise, I was apprised of the existence of ulceration of the cervix uteri. The outer surface of the oss was, as indicated by the touch, smooth, regular, and of a healthy appearance.

Well up the cervix, entirely beyond the reach of the finger, a small granular white spot was observed, about the size of a pea. This was afterwards, upon dilation of the os externum, found to be the inferior border of a large ulcer, extending well up the canal. The despondency of both patient and physician were at once changed into a lively hope, and the patient was at once assured that fears need no longer be entertained of her future health and speedy recovery. The treatment in this case, from this time, consisted mainly in topical applications to the ulcerated surface; nitrate of silver, and nitrate of mercury were the most important, followed in time with free ablutions, consisting of the ferric alum salutum, a pill consisting of extract of belladonna and opii pulv., each one grain: one was wrapped in cotton wool and introduced into the vagina, and was followed with a very happy result, in quieting the stomach. After the first depressing effects of the topical application had passed off, all symptoms assumed a more favorable aspect, and in due time, patient was discharged convalescent.

The next important case, in this connection, was that of a lady aged thirty-eight; bilious temperament; has been troubled with pain for some time in lumbar region, with slight leucorrhœal discharges in last six months; is now having excessive hæmorrhage from vagina.

Having delivered this same lady some four weeks prior to this time, of a three month's conception, and as the placenta, liquor amnie, and fœtus, all came away intact, there was no reason to suppose that the hæmorrhage was dependent upon the common cause, retained placenta. A thorough digital exploration of the vagina was also made in this case, anticipating that by this means I would readily trace the hæmorrhage to an adventitious growth in or about the neck of the uterus; but in this, likewise, I was disappointed. The os, so far as could be reached by the finger, was smooth and regular, but dilated, so that I could readily detect the blood flowing, as I then thought, from within the uterus. The most natural course indicated at this time, seemed to be to treat the case upon general principles, and trust to time for developments of facts. Without following this case further, let me say, in short, that every means and appliance from which I could anticipate the least profit were used. Astringents, arterial opiates and sedatives, and finally the tampon, first well soaked in a strong solution of saccharum saturni and opii, all of which were but temporary in controlling the hæmorrhage. The non-coagulability of the blood, which appeared at an early period, rendered the tampon almost entirely ineffectual, and in spite of all efforts to the contrary the bleeding continued, at times greater, at others less, until patient became almost as pale and pulseless as death. The bleeding had become of a thin, senous character, and death by syncopacy strongly indicated.

Being confident, at that time, that through the touch I could detect any lesion within its reach from which the hæmorrhage would likely proceed, and relying implicitly upon the evidence furnished thereby, together with the assurance from all authority at hand of the extreme infrequency of uterine hæmorrhage only as a result of causes entirely remote from those I could have the least anticipation of disclosing through the speculum, I had treated the case thus far without its use. But as the condition had now become desperate, and having exhausted every available means, as a dernier resort, the speculum was now used, with a vain hope of its disclosing some fact that would aid in the diagnosis and treatment of the case. A bivalve one was selected and used, with the following results. The first observation seemed to simply confirm the deductions made from the previous digital examination. The os was smooth, and, to all outward appearances, healthy, excepting the dilation, which, as before, I supposed to be the result of the excessive bleeding. Those impressions, however, were scarcely made, when my mind was attracted to the extreme elongations of the cervix. This fact was contemplated with some hope that perhaps the superior part of the canal had not been reached by the finger. In this anticipation I was fully requited.

The closed blades of a long pair of caustic-holders was then passed into the cervical canal, at least two inches, without the least resistance. By separating the handles of this instrument the lips were separated, exposing to full view the whole course of the canal, and to my great comfort, the true, unmistakable source and cause of the bleeding being far up the left side of the cervix, and from the vessels involved in an ulcer. Of all men living, it belongs to the physician alone to appreciate the degree of responsibility, the mental physical anxiety and toil . experienced in the treatment of a case like this, and the relief furnished in this examination. And surely it belonged to the light of the speculum alone to set at defiance all future controversy respecting the cause and source of this bleeding, as through it was brought into view almost the very arteries and veins, out from which was fast flowing the life of this patient. A pledget of lint was at once made, around which was tied a silken cord of length sufficient to extend without the vulva. After being well soaked in a solution of per sulphate of iron, this pledget was firmly pressed against the bleeding surface within the canal. In the course of half an hour, the bleeding, for the first time, was completely arrested. In forty-eight hours this pledget was removed by gentle traction at the cord, and a new one inserted. After its removal, the nitrate of silver was topically applied to the ulcer. Sulphate of copper and the ferric alum solutions were used as indicated. From and after the time the first pledget of lint was used, there was no further difficulty experienced in controlling the hæmorrhage; the ulcer yielded kindly to above treatment, and in due time this patient was also discharged, in the enjoyment of perfect health.

That a perfect knowledge of the true pathological condition, and the successful treatment was, in these two instances, dependent upon the use of the speculum, certainly no one will pretend to deny, and doubtless its use, with equal certainty and importance, has been indicated, in many instances, in the practice of almost every physician.

Why not, then, let the speculum at once and forever be regarded as a fixed scientific means of examination of all diseases belonging to this class, and place it in the list where it belongs, among the most important, reliable, and, when used with prudence, as little objectionable an instrument as is known to the profession?

And when considered in the light of its anatomical and physiological predisposition to disease, with the liability to which its position in the body expose it to physical contusions, together with the unlimited abuse which the false and criminal pride and practices of this modern age have subjected it, it cannot be regarded as a far-fetched or unreasonable conclusion that inflammation and ulceration of the cervix uteri is of so very frequent occurrence. Indeed, these deductions are but the re-utterance of facts which are corroborated in the teachings and practice of wise physicians of all ages. And does it not seem reasonable, at least in this nineteenth century—an age in which medicine in all its parts is supposed to be a perfect science—that these plain facts, supported as they are by the experience and observation of the most worthy physicians, from the earliest history of medicine, should now be established, and entitled to full credulity?

But to the contrary, the tidal wave in medicine moves on, and these simple, plain truths, are still the theme of ardent and zealous controversy, some supporting, others utterly denying the existence of ulceration at all, and still others who regard it as the parentage of almost all diseases to which the female constitution is liable.

This demoralized status of this, as well as all other branches of our profession, will ever continue, just so long as every child in medicine assumes the high prerogative of a medical teacher, and their teachings, with every false conception which flits across the medical mind, are permitted to flood the country as high medical authority. Let the proper authorities close up to these premature and chameleon-colored teachers the various avenues through which they reach the public and medical mind. Deprive them of this garb of credulity. Let the inventions of pessaries, and silver-mounted uterine supporters, and in fact all the vast array of cunning inventive genius be subjected to the test of good practical sense, rather than the beautifully-worded recommendations upon which much greater reliance is placed than upon the intrinsic merits of the inventions themselves. Swifter than with the wings of the morning have we been gliding down the stream of medical science, constantly accumulating and gathering, upon every hand, new theories and remedies, until the present. And we now wake up, not to find a well-regulated and accredited science, but to find well-nigh the whole system of medicine submerged beneath a flood of theories both true and false.

Facts which ought to stand out in bold simplicity, are prostrated in utter confusion and turmoil, and is it not a grave and humiliating fact, notwithstanding all our past experience and boasted acquisition of knowledge, that even this branch is still regarded as a mere conjectural art. Are there not truly the greatest responsibilities resting upon the physicians of the present age? And we need not pause a great while in amazement or wonder, contemplating these facts. They are not, by any means, inexplicable.

The normal condition of all well-organized bodies is equally dependent upon actions diametrically opposite in growth and development, disintegration and separation. Let the depurating organs of the human system be closed up for the shortest conceivable time, and notwithstanding the greatest supply of pure, fresh, healthy nutriment is forced into it, yet the inevitable result is disorder and death, and no one for one moment would regard the result as strange or inconsistent.

And none the less dependent is the healthy condition of science upon these same essential vital actions.

Had we not better pause awhile, at this advanced age, and perhaps a careful study of these truths may disclose the very important fact that these vital and reciprocal actions have been greatly neglected, thereby permitting all the rubbish, effete and worn-out material of the great past to accumulate in immeasurable proportions around the very heart of this, as well as every other branch of medicine.

